# Current Research and Development in Hyperthermic Intraperitoneal Chemotherapy (HIPEC)—A Cross-Sectional Analysis of Clinical Trials Registered on ClinicalTrials.gov

**DOI:** 10.3390/cancers15071926

**Published:** 2023-03-23

**Authors:** Kristjan Ukegjini, Marisa Guidi, Kuno Lehmann, Krisztian Süveg, Paul Martin Putora, Nikola Cihoric, Thomas Steffen

**Affiliations:** 1 Department of Surgery, Kantonsspital St. Gallen, 9007 St. Gallen, Switzerland; 2 Department of Visceral and Transplant Surgery, University Hospital Zurich, 8091 Zurich, Switzerland; 3 Department of Radiation Oncology, Kantonsspital St. Gallen, 9007 St. Gallen, Switzerland; 4 Department of Radiation Oncology, Inselspital, University of Bern, 3010 Bern, Switzerland

**Keywords:** peritoneal malignancy, hipec, cytoreductive surgery, intraperitoneal chemotherapy

## Abstract

**Simple Summary:**

Peritoneal metastases have a poor prognosis, and one potential treatment option is cytoreductive surgery (CRS) with hyperthermic intraperitoneal chemotherapy (HIPEC). The specific role of HIPEC is still poorly defined. In this cross-sectional study, we systematically analyzed all HIPEC trials registered on ClinicalTrials.gov to identify current research areas and to provide a perspective on expected outcomes. Only 11% (*n* = 26) of HIPEC trials registered on ClinicalTrials.gov (*n* = 234) have been published. The registered trials are very heterogeneous regarding methodological approaches and study designs. Currently, research is being conducted on 20 different drugs. The most studied cancers in HIPEC trials are peritoneal metastatic colorectal tumors, gastric cancer, and ovarian cancer.

**Abstract:**

Introduction: Over the past two decades, cytoreductive surgery and HIPEC has improved outcomes for selected patients with peritoneal metastasis from various origins. This is a cross-sectional study with descriptive analyses of HIPEC trials registered on ClinicalTrials.gov. This study aimed to characterize clinical trials on HIPEC registered on ClinicalTrials.gov with the primary objective of identifying a trial focus and to examine whether trial results were published. Methods: The search included trials registered from 1 January 2001 to 14 March 2022. We examined the associations of exposure variables and other trial features with two primary outcomes: therapeutic focus and results reporting. Results: In total, 234 clinical trials were identified; 26 (11%) were already published, and 15 (6%) trials have reported their results but have not been published as full papers. Among ongoing nonpublished trials, 81 (39%) were randomized, 30 (14%) were blinded, *n* = 39 (20%) were later phase trials (i.e., phases 3 and 4), *n* = 152 (73%) were from a single institution, and 91 (44%) had parallel groups. Most of the trials were recruiting at the time of this analysis (75, 36%), and 39 (20%) were completed but had yet to publish results. In total, 68% of the trials focused on treatment strategies, and 53% investigated the oncological outcome. The most studied neoplasms for HIPEC trials were peritoneally metastasized colorectal cancer (32%), gastric cancer (29%), and ovarian cancer (26%). Twenty different drugs were analyzed in these clinical trials. Conclusions: Many study results are awaited from ongoing HIPEC trials. Most HIPEC trials focused on gastric, colorectal, or ovarian cancer. Many clinical trials were identified involving multiple entities and chemotherapeutic agents.

## 1. Introduction

Peritoneal metastasis has a dismal prognosis despite systemic treatment [1]. Treatment options for peritoneal malignancy include systemic chemotherapy and, in selected cases, cytoreductive surgery (CRS), with hyperthermic intraperitoneal chemotherapy (HIPEC) providing excellent outcomes in several cohort studies [2,3,4,5]. In theory, CRS is performed to treat macroscopic tumor lesions, and HIPEC is used to treat microscopic residual tumors within a curative treatment regimen [5,6,7,8,9,10]. Despite excellent results for CRS/HIPEC, the specific role of HIPEC remains poorly defined, and recent publications have fueled the ongoing debates about CRS and HIPEC [11,12,13,14,15].

To the best of our knowledge, no published study has empirically analyzed registered clinical trials examining HIPEC. ClinicalTrials.gov was created due to the Food and Drug Administration Modernization (FDAMA) Act of 1997 and was made available to the public in February 2000. The use of registries such as ClinicalTrials.gov has been embraced by the International Committee of Medical Journal Editors (ICMJE). Since 2005, the ICMJE has required trial registration before participant enrollment as a prerequisite for publication in any of its member journals [16]. ClinicalTrials.gov is the largest clinical trial registry, and the registration process and its potential for an analysis of the landscape of clinical trials are well described [17,18,19]. Herein, we quantify the characteristics of HIPEC trials registered on ClinicalTrials.gov to identify the current research fields, identify early discontinuation, report results, and generate an outlook on expected future results.

## 2. Materials and Methods

### 2.1. Selection of Clinical Trials

**Figure 1:**
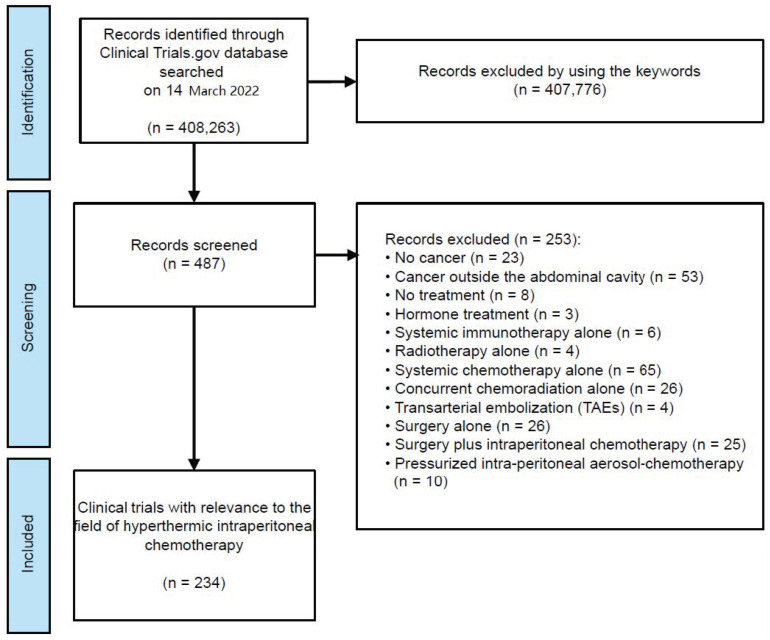
Flow diagram of search for trials from ClinicalTrials.gov, adapted from the Preferred Reporting Items for Systematic Reviews and Meta-Analyses (PRISMA) statement.

We downloaded an XML data set comprising all 408,263 clinical trials registered with ClinicalTrials.gov on March 14th, 2022. Our analysis was restricted to HIPEC trials. To identify potential HIPEC trials, we searched for the terms: “intraperitoneal chemotherapy”, “hipec”, “hyperthermic intraperitoneal chemotherapy”, “peritoneal metastases”, “peritoneal carcinomatosis”, “peritoneal malignancy”, and “cytoreductive surgery”. This search strategy resulted in 487 (100%) trials for manual review and classification. All trials addressing diseases other than cancer, treatment with pressurized intraperitoneal aerosol chemotherapy (PIPAC), intraperitoneal chemotherapy (IP) delivery via a catheter, or different types of therapy were excluded from this analysis. The remaining 234 (48%) trials were included in our analysis. The trial selection process is shown in Figure 1.

### 2.2. Data from ClinicalTrials.gov

Data on the trial characteristics, registration, completion of the trials, study design, enrollment characteristics, funding source, and number of study sites for all clinical trials were extracted from the ClinicalTrials.gov.

Included trials were divided classified by (1, 1/2, 2, 2/3, 3, 4, NA): the number of participants; allocation status (randomized vs. nonrandomized); trial start year; region; intervention type (drug, procedure, biological, behavioral, device, dietary supplement, diagnostic test, radiation, combination product, other); primary purpose (treatment, prevention, diagnostic, supportive care, other); overall status and primary outcome (efficacy, safety, feasibility, pharmacodynamics/pharmacokinetics, quality of life, other).

To determine the trial focus, each included clinical trial was classified according to its primary disease origins: peritoneal metastases from gastric cancer, colorectal cancer, cancer from the hepatopancreatic origin, ovarian cancer, primary peritoneal malignancy, etc. Each trial was assigned to 1 or multiple appropriate categories of disease origin.

All clinical trials on solid-organ tumors were manually reviewed to evaluate the therapeutic focus, and each trial was assigned to one or more appropriate drug categories. One or more therapeutic foci per study were accepted. For the analyses, each therapeutic focus was treated as a binary variable. All reported interventions were evaluated and classified into the following therapeutic groups: experimental and approved drugs, based on the development status of the specific therapy. Experimental drugs were annotated as such if no previous indication was approved for commercial use by the United States Food and Drug Administration

The trials were classified according to the reported experimental intervention types. If randomization was not explicitly defined, a manual review of the trial entries was performed to determine the study design.

Funding sources include foundations (e.g., National Institutes of Health, government networks), industry, and academic institutions (e.g., universities, hospitals, foundations, and other nonprofit organizations).

Frequencies and percentages were provided for categorical characteristics; medians and interquartile ranges (IQRs) were provided for continuous characteristics.

### 2.3. Search for Publications of Trial Results

To identify if trials registered at ClinicalTrials.gov were published, the electronic databases EMBASE, MEDLINE (via PubMed), and Cochrane Central Register of Controlled Trials were searched. We included the trial registration number (NCT number) in the search for publications searching for given trial characteristics. If more than one publication was identified, we chose the publication that most closely fit the study described in the record. We then searched an online result registry ClinicalStudyResults.org (accessed on 25 October 2022) and result reports available through company Web pages for references to publications [20,21]. 

We defined a trial as published if the primary outcome(s) results were published in a peer-reviewed journal. We also recorded whether the trial was reported elsewhere in other publication types, such as articles without results presentation, conference abstracts, clinical study reports, and records in trial registries.

Bibliometric data of the journals where the HIPEC trials were published were found in the Journal Citation Reports of the ISI Web of Knowledge [22]. Citation reports were extracted for the individual papers from the Web of Science for the main publication from each trial [23].

### 2.4. Outcome Parameters

The primary outcomes were the trial’s focus (colorectal carcinoma, ovarian carcinoma, appendiceal carcinoma, etc.) and trial completion. The study completion date is defined by ClinicalTrials.gov as the date when participants are no longer being examined or treated (last patient’s final visit). Each record was classified as including study results in links to PubMed abstracts, links to unpublished result reports, or actual study results.

Secondary outcomes were characteristics of registered clinical trials. Data on characteristics of the trials and their registration include registration date, start date, completion date, condition treated, funding source, trial phase, primary outcome, anticipated enrolment number, age group of participants, and elements of the study design. Two authors (K.U., M.G.) independently performed the literature search and data extraction, and consensus resolved disagreements.

### 2.5. Ethical Statement

This study was reviewed by the Ethical Committee of the Cantonal Hospital St. Gallen and was exempt from oversight and informed consent because all data are publicly available. Our findings are reported in accordance with the Strengthening the Reporting of Observational Studies in Epidemiology (STROBE) reporting guidelines.

## 3. Results

The characteristics of all trials for HIPEC registered in ClinicalTrials.gov are shown in Table 1. In total, 64% of the clinical trials were small in terms of the number of participants (100 or fewer). Overall, 96% of these trials had an anticipated enrollment of 500 or fewer participants, and the median number of participants per trial was *n* = 60 (IQR, 1–15,000). Most trials (*n* = 158, 68%) focused on treatment strategies, *n* = 123 (53%) trials investigated the overall survival, and *n* = 130 (56%) trials analyzed drugs. Most of the trials received academic funding (195, 83%). Most trials were performed at single sites (*n* = 170, 73%); *n* = 64 (27%) were multisite trials. Most trials were initiated in a European or North American research site (*n* = 34% vs. 32%). A few trials (*n* = 52, 22%) were restricted to women, a difference mainly attributable to ovarian cancer trials. There were also differences in age distribution among the three categories. Most trials included only adult patients. Eight trials had been withdrawn: four were withdrawn because of poor accrual rate, two were withdrawn because the study was not funded, and two were withdrawn because of logistical problems before the recruitment phase.

Several missing data elements exist for some characteristics: 19% of the trials were missing data regarding the primary purpose; 5% of the trials did not report the intervention type; 9% of the trials did not report the number of institutions or the region; 34% of the trials did not report the randomization method or trial phase; and 15% of the trials did not report the overall status.

Of the 234 clinical trials we reviewed, *n* = 26 (11%) were already published, and *n* = 15 (6%) trials had reported their results elsewhere. Some studies were published in high-ranked journals, such as The New England Journal of Medicine (NCT00426257) [24], JAMA Surgery (NCT01091636) [25], Lancet Oncology (NCT00769405, NCT01226394, NCT01835041) [26,27,28], or the Lancet Gastroenterology Hepatology (NCT02231086) [29]. Most trials were published in the Annals of Surgical Oncology (NCT00458809, NCT02891447, NCT03230240, NCT02575859, NCT02863471) [30,31,32,33,34].

The cancer entity involved in the trial had a significant impact on citation scores: papers on ovarian cancer and colorectal cancer research resulted in many more citations than those on other cancer types. European countries consistently published the most articles (*n* = 14), followed by the United States (*n* = 7), which had a relatively high commitment to surgical oncology research. The output of East Asian countries (China, Republic of Korea, Singapore, Taiwan) has been increasing rapidly over time but is still below that of the larger European countries. Some data elements for some characteristics are missing or not added or updated in a total of 23% of published trials reported the incorrect overall recruitment status; 31% of published trials did not report the randomization methods; 31% of trials did not report the phase; and 26% of trials did not report their primary endpoint.

Among ongoing nonpublished trials, *n* = 170 (82%) were interventional trials, *n* = 81 (39%) were randomized, *n* = 30 (14%) were blinded, *n* = 39 (20%) were later-phase trials (i.e., phases 3 and 4), *n* = 152 (73%) were from a single institution, and *n* = 91 (44%) had parallel groups. Most of the trials were recruiting at the time of this analysis (75, 36%), and 39 (20%) were completed but had not published results on the primary endpoint (shown in Supplementary Table S1).

A detailed analysis of the relative commitment to ongoing HIPEC research at cancer sites and an analysis of the investigated agents are displayed in Table 2. Multiple tumor types were analyzed in *n* = 90 of 208 ongoing trials. Thirty-three of the *n* = 208 ongoing trials included patients with either primary or secondary peritoneal metastases, regardless of the primary tumor (colorectal carcinoma, ovarian carcinoma, appendiceal carcinoma, etc.). The most widely studied neoplasms for HIPEC trials were peritoneal metastasized colorectal cancer (*n* = 67, 32%), peritoneal metastasized ovarian cancer (*n* = 61, 29%), and peritoneal metastasized gastric cancer (*n* = 54, 26%). Neoplasia sites with smaller percentages of HIPEC clinical trials were peritoneal metastasized liver cancer, peritoneal metastasized gallbladder cancer, and peritoneal metastasized bladder cancer (*n* = 1 (0.4%), *n* = 1 (0.4%), and *n* = 1 (0.4%), respectively).

Twenty different drugs (cisplatin, mitomycin- C, irinotecan, doxorubicin, paclitaxel, oxaliplatin, 5-fluorouracil, docetaxel, lobaplatin, carboplatin, anti-PD-1 antibody, thalidomide, leucovorin, melphalan, MOC31PE immunotoxin, capecitabine, raltitrexed, pasireotide, gemcitabine, and cantrixil) were analyzed in these clinical trials. The drugs with the highest proportions of clinical tests were cisplatin (*n* = 78 (33%)), mitomycin C (*n* = 69 (29%)), and oxaliplatin (*n* = 37 (17%)) (shown in Table 2).

The phase III trial characteristics are shown in Table 3. Thirty-nine phase III and one phase IV ongoing trials are registered at ClinicalTrials.gov. Of the 20 different tumor entities analyzed in the ongoing trials, the phase III and phase IV trials focus on gastric carcinoma (*n* = 13), ovarian carcinoma (*n* = 12), and colon carcinoma (*n* = 11).

Fourteen ongoing later-phase clinical trials (i.e., phases II/III and III) evaluated the efficacy and safety of HIPEC in the treatment of patients with peritoneal metastases of gastric origin (shown in Table 2). HIPEC is analyzed for two indications in gastric cancer: first, as a definitive treatment in established peritoneal metastases (NCT03348150, NCT03179579, NCT03023436, NCT02356276, NCT02158988, NCT00052962); second, as an adjuvant therapy after curative surgery for patients with locally advanced gastric cancer and a high risk of developing peritoneal metastases (NCT04597294, NCT04447352, NCT03917173, NCT02960061, NCT02381847, NCT02240524, NCT01882933, NCT01683864) (shown in Table 3).

Three phase III ongoing clinical trials (NCT01628380, NCT03772028, NCT03842982) evaluated the efficacy of HIPEC for advanced-stage ovarian cancer used in an adjuvant setting and one phase III clinical trial (NCT03180177) in neoadjuvant setting patients. HIPEC was analyzed in two phase III trials (NCT03373058 and NCT02328716) as a definitive treatment in ovarian cancer with established peritoneal metastases. Three phase III clinical trials (NCT01376752, NCT03220932, NCT03371693) have the primary objective of comparing the efficacy and safety of CRS alone versus CRS plus HIPEC of first or second platinum-resistant ovarian cancer recurrence (shown in Table 3).

Fourteen ongoing later-phase research clinical trials (i.e., phases II/III, III and IV) evaluated the efficacy and safety of HIPEC in the treatment of patients with peritoneal metastases of colorectal origin (shown in Table 2). Six randomized phase III trials studied HIPEC for patients with locally advanced colorectal cancer and a high risk of developing peritoneal metastases in an adjuvant mode in addition to standard treatment to prevent the development of peritoneal metastasis (NCT02179489, NCT02965248, NCT02974556, NCT03221608, NCT03914820 and NCT04370925). One phase IV clinical trial (NCT05250648) evaluated the effectiveness of HIPEC with high-dose mytomicin-C in preventing the development of peritoneal recurrence in patients with limited peritoneal metastasis from colon cancer (not rectal) after CRS (shown in Table 3).

**Figure 2:**
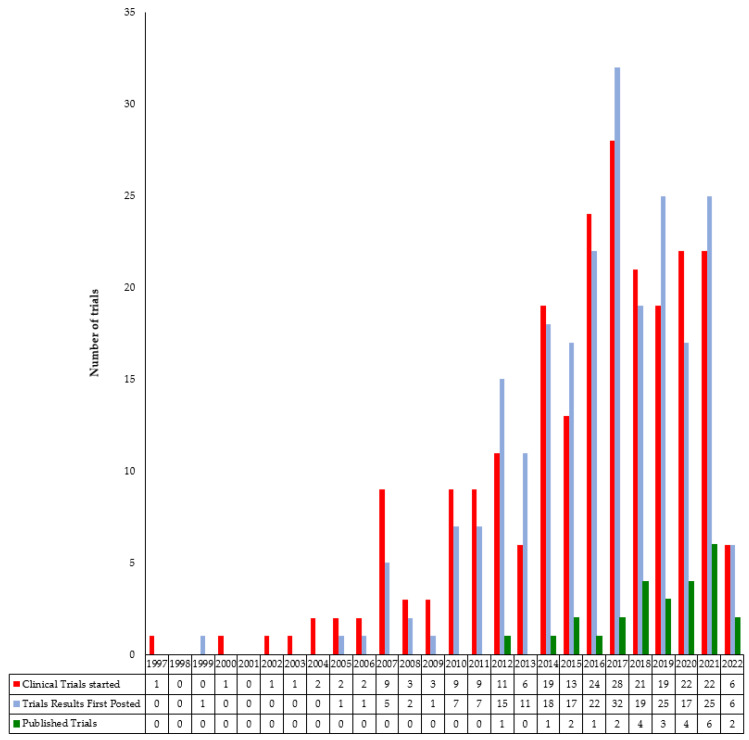
Cumulative number of clinical trials that started per year, that reported results to ClinicalTrials.gov, or that were published.

The annual number of clinical trials in the field of HIPEC that started per year worldwide or reported results to ClinicalTrials.gov is shown in Figure 2. The three curves are almost parallel. Reporting trial results (journal publications and other reports included) was more common after 2010. The number of trials submitted for registration in ClinicalTrials.gov increased over the two periods: from *n* = 25 in 1997–2009 to *n* = 203 in 2010–2021. The mean time between the estimated end of the study and the first publication of results was four years (range 0.1–15 years). However, the end of each survey could not be analyzed herein.

## 4. Discussion

### 4.1. Main Findings

In this study, we estimated that only 11% of HIPEC trials registered at ClinicalTrials.gov were published. Compared to other research fields, CRS/HIPEC is poorly represented. Between 1997 and 2022, only 234 clinical trials examining HIPEC were registered. Many publications on HIPEC are expected in the following years. HIPEC trials increased at a significant rate from 2010. Registered trials are highly heterogeneous in terms of methodological approaches and study designs. Research is currently being performed with 20 different drugs, including experimental drugs. Small, single-center clinical trials on HIPEC dominate the ClinicalTrials.gov database, but most are funded by academic institutions or cancer foundations. The most studied cancer entities in HIPEC trials are peritoneal metastasized colorectal, gastric and ovarian cancers.

### 4.2. Results in Context

With the concept of CRS combined with HIPEC, many encouraging results were found in randomized controlled trials and large cohort studies. The introduction of CRS and HIPEC in the 1990s, but especially after 2000, has obtained unprecedented results in patients with peritoneal metastases of colorectal cancer; thus, it has gradually been accepted, even being considered the best treatment for these patients [35,36,37,38,39]. In the following years, more trials were registered. Clinical research on HIPEC represents a key component of the multiple efforts needed to reduce the disease burden in patients and to advance the treatment of peritoneal metastases. The progress of the field warrants and partially depends on a greater commitment of resources and funding by all clinical trial sponsors. In addition to increasing the number of HIPEC clinical trials, improvement in HIPEC trial completion and dissemination is important. We found a mean delay of 4 years between study completion and publication. Scarce reports of study results, particularly in academic-funded trials, may reflect relatively limited resources in academia. Although federal statutes require many clinical trials to report their results within one year with the option to delay for 2 years [40], the parameters of the statutes have evolved over the past decade, and barriers exist to consistent results reporting [41,42,43,44]. A lack of results can bias the literature, squander limited resources, and hinder medical innovation. Greater results reporting within HIPEC trials continues to be a relevant goal, with implications for all physicians who treat patients with peritoneal metastases.

In treating primary peritoneal cancer and the pseudomyxoma peritonei and the treatment of peritoneal metastasized colorectal, ovarian, or gastric cancer, the addition of HIPEC has been used in specialized centers worldwide. HIPEC was introduced and performed for peritoneal malignancies worldwide. However, despite its proliferation, the procedure still needs to be better studied, and execution of solid large multicentric randomized trials remains difficult. Many scientific projects were terminated early because of difficulties in recruiting. Heterogeneity in chemotherapeutics and technical aspects of HIPEC, as well as many different therapeutic concepts, have hindered the comparability of studies.

The French PRODIGE-7 trial (NCT00769405) [26] has most recently cast doubt on the benefit of HIPEC. The authors found no overall survival benefit by adding HIPEC to cytoreductive surgery and more frequent postoperative late complications, suggesting that CRS alone should be the cornerstone of therapeutic strategies. These study results are still controversial today. Additionally, prophylactic HIPEC in so-called high-risk situations in patients with colorectal cancer has lost popularity after the Dutch and French RCTs were published. In ovarian cancer, a Dutch study by Van Driel et al. [24] showed promising results in patients with stage III epithelial ovarian cancer with more prolonged recurrence-free survival and overall survival than surgery alone. Despite an unclear picture after the publication of the most recent trials, the rationale of CRS and HIPEC remains logical to many surgeons and medical oncologists. The ongoing studies, hopefully, provide more clarity in the field. Six ongoing randomized, phase III trials studied HIPEC for patients with locally advanced colorectal cancer and a high risk of developing peritoneal metastases in an adjuvant mode in addition to standard treatment to prevent the development of peritoneal metastasis (NCT02179489, NCT02965248, NCT02974556, NCT03221608, NCT03914820 and NCT04370925). Probably the most important ongoing study on this topic is the GECOP-MMC-Trial (NCT05250648). It is a prospective, open-label, randomized, multicenter phase IV clinical trial that evaluates the effectiveness of HIPEC with high-dose mytomicin-C in preventing the development of peritoneal recurrence in patients with limited peritoneal metastasis from colon cancer (not rectal), after CRS [45].

### 4.3. Strengths/Limitations

Our analysis uniquely examines the therapeutic focus of HIPEC trials, publication status, and study characteristics. This analysis allows for extrapolation of factors that may contribute to the low number of HIPEC trials and increased risk of premature discontinuation in HIPEC trials. We present novel assessments of temporal trends that are only possible with a large sample and a longer period. This study examines the drugs used to treat peritoneal metastases and reviews and compares reporting of results from ongoing trials.

The present cross-sectional analysis has some limitations. First, the ClinicalTri-als.gov registry represents only a sample of all global clinical trials; there are several other registries (e.g., European Union Clinical Trials Registry (EudraCT) or Clinical Trials Registry India (CTRI)) that can also be used worldwide. Trials are registered in one of the other registries and therefore were not examined in our study. However, a study found that ClinicalTrials.gov contains the greatest number of trials compared with other data-bases [46].

Second, there were changes in the data collected, the definitions used, and the rigor with which missing data were tracked. Due to practical or logistical limitations, some data elements needed to be included or available. 

Third, because this analysis involves multiple testing, it is possible that the strength of association seen for some trial features may be the result of chance. 

Fourth, the data sets for all problems in the database are probably only sometimes complete and up to date. Some of these trials may have been in the pre-commercialization phase or were early negative studies for which plans for commercialization were withdrawn. Because sponsors submit study characteristics, verifying their accuracy is impossible. 

Fifth, during data collection, certain data may have needed to be misclassified during selection and classification or some studies were registered incorrectly. We greatly minimized these limitations: Two authors (K.U. and M.G.) cross-checked all identified studies and the selection steps. Finally, the limitations of ClinicalTrials.gov have been described in other studies and applied to this analysis [47,48].

### 4.4. Implications for Future Research

Based on our results, we identified a broad heterogeneity of drugs used for HIPEC for different tumor entities, which hinders the development of comparable study designs. Inclusion criteria for patients in future studies should be defined very clearly. Otherwise, study results may be flawed, as in the abovementioned French study by Quenet et al. [26]. The range of primary tumor types for peritoneal metastases and additional histologic subtypes in general is too broad and therefore does not allow for the generalization of treatment options and indications for HIPEC. This applies under the study conditions. 

A critical implication for future research is clearly the need for better multicentric studies in the field of HIPEC. Such studies should be performed according to study parameters and stratifications that need to be better defined and yet to be developed. 

### 4.5. Implications for the Practice

It is essential to participate in ongoing and future multicentric trials rather than performing another large cohort trial over a long period with different HIPEC drug regimens or heterogeneous patient groups. Systematic chemotherapies have developed dramatically over the past years with excellent results. Therefore, the benefit of HIPEC might have been shrinking in parallel, making it more challenging to detect the solid survival advantages of HIPEC. HIPEC should be performed in experienced centers and whenever possible under study conditions. Sixty-seven ongoing clinical trials are evaluating the efficacy and safety of HIPEC in treating patients with peritoneal metastases of colorectal origin. Most likely, the most important ongoing study on this topic is the GECOP-MMC-Trial (NCT05250648). This prospective, open-label, randomized, multicenter phase IV clinical trial evaluates the effectiveness of HIPEC with high-dose mitomycin C in preventing the development of peritoneal recurrence in patients with limited peritoneal metastasis from colon cancer (not rectal) after CRS [45]. There is a broad heterogeneity of drugs used for different tumor entities of up to twenty different chemotherapeutics used for HIPEC in ovarian cancer. There is an urgent need for clinical guidelines on the administration and type of drugs to be used.

On the other hand, clinicians should, whenever possible, adopt existing clinical recommendations; an early example is the ASPSM scheme published in 2013 [49].

Currently, 39 phase III and one phase IV ongoing trials are registered on ClinicalTrials.gov. Of the more than 20 tumor entities analyzed in ongoing trials, phase III and IV trials focus mainly on colonic, gastric, and ovarian cancers. Twenty-eight ongoing randomized controlled phase III trials and one phase IV trial evaluated the efficacy and safety of HIPEC in an adjuvant setting for cancer patients with peritoneal dissemination. The primary aim of this study was to compare the overall survival and disease-free survival between cancer patients with limited peritoneal carcinomatosis and/or tumor-positive peritoneal cytology treated with cytoreductive surgery and HIPEC and those treated with the current standard treatment.

To date, there is also a discrepancy in how patients with ovarian cancer recurrence should be treated. Most patients with recurrences are currently treated with new combinations of systemic chemotherapy. Repeated laparotomy with complete cytoreduction is an option. Two phase III prospective randomized trials (NCT04473339, NCT01376752) compared cytoreductive surgery with or without HIPEC, and one phase III trial (NCT03220932) compared cytoreductive surgery with HIPEC versus chemotherapy alone.

Neoadjuvant therapy concepts with HIPEC were recently discussed, and one phase III multicenter, prospective, randomized controlled clinical trial evaluated the efficacy of HIPEC used in the neoadjuvant setting for advanced-stage epithelial ovarian cancer patients eligible for CRS before planned surgery (NCT03180177). Two phase II (NCT04308837, NCT05095467) and one phase III (NCT03179579) trials evaluated the efficacy of a multimodality approach to treating patients with locally advanced gastric cancer by incorporating diagnostic laparoscopy with HIPEC in a neoadjuvant setting followed by surgical resection and adjuvant chemotherapy. The trial aims of studies with a multimodality approach are inducing pathological complete response; rates of disease progression during neoadjuvant therapy; and overall, disease-free and peritoneal disease-free survival. A phase II single-center, prospective proof-of-concept study (NCT02850874) evaluated the surgical outcomes and clinicopathologic results of neoadjuvant HIPEC in conjunction with perioperative systemic chemotherapy (neoadjuvant and adjuvant) and pancreaticoduodenectomy in a small cohort of patients having T1-T3 resectable pancreatic ductal adenocarcinoma with one or more high-risk clinical features.

## 5. Conclusions

Many study results from ongoing HIPEC trials are expected because only a small percentage of HIPEC trials registered at ClinicalTrials.gov have been published. Most trials on HIPEC are focused on gastric, colorectal, or ovarian cancers. A large heterogeneity in terms of methodological approaches and study designs of clinical trials was identified involving multiple entities and chemotherapeutic agents.

## Figures and Tables

**Table 1:** Characteristics of HIPEC trials registered in ClinicalTrials.gov.

Characteristics	All Trials	Journal Publications	Not Published in Journals	
	*n* = 234 (100%)	*n* = 26 (11%)	*n* = 208 (89%)	
			Results Reported Elsewhere	No Results Available
			*n* = 15 (6%)	*n* = 193 (83%)
Primary purpose of trial				
Treatment	158	18	12	128
Prevention	13	1	0	12
Diagnostic	5	0	0	5
Supportive care	5	0	0	5
Other ^a^	9	1	2	6
Missing	44	6	1	37
Intervention type ^b^				
Drug	130	16	10	104
Procedural	114	13	9	92
Biological	3	0	0	3
Behavioral	5	1	0	4
Device	9	0	0	9
Dietary supplement	1	0	0	1
Diagnostic Test	6	0	0	6
Radiation	3	0	0	3
Combination Product	3	0	1	2
Other	33	4	5	24
Missing	12	0	0	12
Funding ^b^				
Academic	195	17	11	167
Industry	5	0	0	5
Cancer foundation	48	11	6	31
Missing	1	0	0	1
Region ^b^				
Africa	2	0	0	2
Australia	1	0	0	1
Europe	79	14	6	59
North America	76	7	9	60
Central and South America	2	1	0	1
Asia and Pacific	53	4	0	49
Middle East	1	0	0	1
Missing	22	0	0	22
Number of institutions/Collaboration				
1	170	18	13	139
2	9	0	1	8
3–10	20	3	1	16
>10	15	1	0	14
Missing	20	4	0	16
Anticipated enrollment, No. of patients				
1–9	4	0	0	4
10–49	88	12	6	70
50–99	57	7	2	48
100–499	75	7	6	62
500–999	7	0	0	7
>1000	2	0	1	1
Missing	1	0	0	1
Sex				
Female only	52	4	4	44
Male only	0	0	0	0
Both	182	22	11	149
Age of study population				
Children only	1	0	0	1
Children and adults	18	0	2	16
Adults only	215	26	13	176
Study type				
Interventional	190	20	14	156
Observational	44	6	1	37
Allocation status ^c^				
Randomized	90	9	7	74
Nonrandomized	20	3	1	16
Missing	80	8	6	66
Interventional group				
Single group	87	10	6	71
Parallel	101	10	8	83
Sequential	1	0	0	1
Missing	1	0	0	1
Blinding ^c^				
None (open label)	157	18	12	127
Single blind	14	2	1	11
Double blind	9	0	0	9
Triple blind	6	0	1	5
Quadruple blind	3	0	0	3
Missing	1	0	0	1
Trial phase				
Phase I	25	2	0	23
Phase I/II	10	2	0	8
Phase II	69	8	6	55
Phase II/III	5	1	2	2
Phase III	44	5	3	36
Phase IV	1	0	0	1
Missing	80	8	4	68
Overall status				
Not yet recruiting	21	0	0	21
Recruiting	75	0	3	72
Completed	63	18	6	39
Suspended	1	0	0	1
Terminated	15	2	5	8
Withdrawn	8	0	0	8
Active, not recruiting	14	3	0	11
Enrolling by invitation	1	0	0	1
Unknown status	36	3	1	32
Length of study conduct				
<1 y	14	3	0	11
1–2 y	29	4	0	25
2–5 y	93	7	6	80
5–10 y	79	11	8	60
>10 y	16	1	1	14
Missing	3	0	0	3
Primary Outcome ^d^				
Efficacy	123	14	9	100
Safety	79	12	4	63
Feasibility	21	1	4	16
Pharmacodynamics/pharmacokinetics	29	4	0	25
Quality of life of patients	9	1	0	8
Other ^e^	37	3	3	31

^a^ Includes health services research and basic science. ^b^ Percentages may not add up to 100%, as categories are not mutually exclusive. ^c^ Only collected for interventional studies. ^d^ Trials could have >1 therapeutic focus. For analysis, each therapeutic focus was treated as a binary variable. ^e^ Includes lab draws (inflammatory parameters, biomarkers etc.), tumoral biopsy tissue, microbiome, and diagnostic.

**Table 2:** Number of clinical trials involving different drugs by neoplasia site.

	Colorectal ^a^ (*n* = 67)	Ovarian ^a^ (*n* = 61)	Gastric ^a^ (*n* = 54)	Primary Peritoneal ^a^ (*n* = 35)	From Each Origin ^a,b^ (*n* = 33)	Appendiceal ^a^ (*n* = 21)	Fallopian Tube ^a^ (*n* = 20)	Non-Carcinoma Tumors ^a,c^ (*n* = 8)	Pancreatic ^a^ (*n* = 7)	Uterine ^a^ (*n* = 5)	Cervical ^a^ (*n* = 3)	Bile Duct Cancer ^a,d^ (*n* = 2)	Small Intestine ^a^ (*n* = 1)	Bladder ^a^ (*n* = 1)
Used drugs, *n* (%) ^e^														
Cisplatin	4 (6)	20 (33)	16 (30)	11 (31)	5 (15)	1 (5)	9 (45)	3 (38)	2 (29)	1 (20)	0 (0)	0 (0)	0 (0)	0 (0)
Mitomycin C	18 (27)	7 (11)	15 (28)	7 (20)	2 (6)	7 (33)	4 (20)	2 (25)	2 (29)	3 (60)	3 (100)	1 (50)	1 (100)	1 (100)
Irinotecan	3 (4)	0 (0)	3 (6)	1 (3)	0 (0)	0 (0)	0 (0)	0 (0)	0 (0)	0 (0)	0 (0)	0 (0)	0 (0)	0 (0)
Doxorubicin	1 (1)	5 (8)	3 (6)	1 (3)	0 (0)	1 (0)	3 (15)	3 (38)	1 (14)	2 (40)	2 (67)	1 (50)	1 (100)	0 (0)
Paclitaxel	1 (1)	8 (13)	18 (33)	2 (6)	1 (3)	0 (0)	3 (15)	0 (0)	0 (0)	0 (0)	0 (0)	0 (0)	0 (0)	0 (0)
Oxaliplatin	13 (19)	2 (3)	6 (11)	4 (11)	0 (0)	3 (14)	0 (0)	0 (0)	0 (0)	0 (0)	0 (0)	0 (0)	0 (0)	0 (0)
5-Fluorouracil	3 (4)	0 (0)	3 (6)	1 (3)	1 (3)	0 (0)	0 (0)	0 (0)	0 (0)	0 (0)	0 (0)	0 (0)	0 (0)	0 (0)
Docetaxel	0 (0)	3 (5)	3 (6)	1 (3)	0 (0)	0 (0)	1 (5)	0 (0)	0 (0)	0 (0)	0 (0)	0 (0)	0 (0)	0 (0)
Lobaplatin	2 (3)	1 (2)	2 (4)	0 (0)	0 (0)	0 (0)	0 (0)	0 (0)	0 (0)	0 (0)	0 (0)	0 (0)	0 (0)	0 (0)
Carboplatin	1 (1)	12 (20)	1 (2)	6 (17)	1 (3)	0 (0)	8 (40)	0 (0)	0 (0)	1 (20)	0 (0)	0 (0)	0 (0)	0 (0)
Anti-PD-1 antibody	0 (0)	0 (0)	1 (2)	0 (0)	0 (0)	0 (0)	0 (0)	0 (0)	0 (0)	0 (0)	0 (0)	0 (0)	0 (0)	0 (0)
Thalidomide	2 (3)	0 (0)	0 (0)	0 (0)	0 (0)	1 (5)	0 (0)	0 (0)	0 (0)	0 (0)	0 (0)	0 (0)	0 (0)	0 (0)
Leucovorin	1 (1)	0 (0)	0 (0)	0 (0)	0 (0)	1 (5)	0 (0)	0 (0)	0 (0)	0 (0)	0 (0)	0 (0)	0 (0)	0 (0)
Melphalan	1 (1)	0 (0)	0 (0)	0 (0)	0 (0)	1 (5)	0 (0)	0 (0)	0 (0)	0 (0)	0 (0)	0 (0)	0 (0)	0 (0)
MOC31PE	1 (1)	0 (0)	0 (0)	0 (0)	0 (0)	0 (0)	0 (0)	0 (0)	0 (0)	0 (0)	0 (0)	0 (0)	0 (0)	0 (0)
Capecitabine	1 (1)	0 (0)	0 (0)	0 (0)	0 (0)	0 (0)	0 (0)	0 (0)	0 (0)	0 (0)	0 (0)	0 (0)	0 (0)	0 (0)
Raltitrexed	2 (3)	0 (0)	0 (0)	0 (0)	0 (0)	0 (0)	0 (0)	0 (0)	0 (0)	0 (0)	0 (0)	0 (0)	0 (0)	0 (0)
Pasireotide	1 (1)	1 (2)	0 (0)	1 (3)	0 (0)	0 (0)	0 (0)	0 (0)	0 (0)	0 (0)	0 (0)	0 (0)	0 (0)	0 (0)
Gemcitabine	0 (0)	1 (2)	1 (2)	1 (3)	0 (0)	0 (0)	1 (5)	1 (13)	3 (42)	1 (20)	0 (0)	1 (50)	0 (0)	0 (0)
Cantrixil	0 (0)	1 (2)	0 (0)	0 (0)	0 (0)	0 (0)	1 (5)	0 (0)	0 (0)	0 (0)	0 (0)	0 (0)	0 (0)	0 (0)
Missing	2 (3)	1 (2)	0 (0)	1 (3)	24 (75)	11 (52)	0 (0)	1 (13)	1 (14)	1 (20)	0 (0)	0 (0)	0 (0)	0 (0)
Allocation status														
Randomized	27 (40)	22 (36)	21 (39)	10 (29)	12 (36)	6 (29)	5 (25)	0 (0)	2 (29)	0 (0)	0 (0)	1 (50)	0 (0)	0 (0)
Nonrandomized	5 (8)	9 (15)	1 (2)	3 (8)	2 (6)	3 (14)	2 (10)	2 (25)	3 (42)	2 (40)	2 (67)	1 (50)	1 (100)	0 (0)
Missing	35 (52)	30 (49)	32 (59)	22 (63)	19 (58)	12 (57)	13 (65)	6 (75)	2 (29)	3 (60)	1 (33)	0 (0)	0 (0)	1 (100)
Trial phase, *n* (%)														
Phase I	7 (10)	11 (18)	4 (7)	7 (20)	1 (3)	2 (10)	5 (25)	3 (37.5)	1 (14)	1 (20)	0 (0)	0 (0)	0 (0)	0 (0)
Phase I/II	4 (6)	2 (3)	3 (6)	3 (8)	0 (0)	0 (0)	1 (5)	0 (0)	0 (0)	0 (0)	0 (0)	0 (0)	0 (0)	0 (0)
Phase II	17 (25)	19 (31)	21 (39)	12 (35)	5 (15)	8 (38)	8 (40)	3 (37.5)	4 (58)	2 (40)	2 (67)	2 (100)	1 (100)	0 (0)
Phase II/III	2 (3)	0 (0)	1 (2)	0 (0)	0 (0)	1 (5)	0 (0)	0 (0)	1 (14)	0 (0)	0 (0)	0 (0)	0 (0)	0 (0)
Phase III	11 (17)	12 (20)	13 (24)	3 (8)	3 (9)	0 (0)	3 (15)	0 (0)	0 (0)	0 (0)	0 (0)	0 (0)	0 (0)	0 (0)
Phase IV	1 (1)	0 (0)	0 (0)	0 (0)	0 (0)	0 (0)	0 (0)	0 (0)	0 (0)	0 (0)	0 (0)	0 (0)	0 (0)	0 (0)
Missing	25 (38)	17 (28)	12 (22)	10 (29)	24 (73)	10 (47)	3 (15)	2 (25)	1 (14)	2 (40)	1 (33)	0 (0)	0 (0)	1 (100)

^a^ The number of trials indicates in how many of the 208 ongoing trials the tumor entity was analyzed, if this is comprehensible. Number of trials may not add up to 208 because trials could use >1 type of neoplasia. For analysis, each type of neoplasia was treated as a binary variable. ^b^ These trials did not differentiate which neoplasia types were involved. ^c^ Histologically proven diffuse peritoneal or retroperitoneal tumor from the following histology: desmoplastic round cell tumor, ovarian germ cell, sarcoma, rhabdomyosarcoma, Wilms’ tumor, late-stage neuroblastoma, or other non-carcinoma tumors. ^d^ Include Gallbladder cancer and cholangiocarcinoma of the extrahepatic bile duct or liver ^e^ Percentages may not add up to 100% because trials could have used >1 drug. For analysis, each drug was treated as a binary variable.

**Table 3:** Ongoing phase III and IV trials.

ClinicalTrials.Gov Trial ID	Phase	Treatment Allocation	Number of Participants	Primary Purpose of Trial	Condition or Disease	Chemotherapy Drugs	Trial Status
NCT00052962	Phase III	Randomized	30	Treatment	Peritoneal carcinomatosis from each origin	Cisplatin	Completed
NCT04981639	Phase III	Randomized	72	Supportive care	Peritoneal carcinomatosis from each origin	N/A	Recruiting
NCT03359811	Phase III	Randomized	75	Treatment	Peritoneal carcinomatosis from each origin	N/A	Completed
NCT03180177	Phase III	Randomized	263	Treatment	Primary peritoneal carcinoma AND Ovarian AND Tube carcinoma	Paclitaxel AND/OR cisplatin	Not yet recruiting
NCT03373058	Phase III	Randomized	310	Treatment	Primary peritoneal carcinoma AND Ovarian AND Tube carcinoma	Docetaxel AND/OR Cisplatin	Recruiting
NCT02328716	Phase III	Randomized	32	Other	Primary peritoneal carcinoma AND Ovarian AND Tube carcinoma	Cisplatin	Unknown status
NCT01628380	Phase III	Randomized	94	Treatment	Ovarian cancer	Cisplatin AND Paclitaxel	Unknown status
NCT02681432	Phase III	Randomized	60	Treatment	Ovarian cancer	Paclitaxel	Unknown status
NCT03220932	Phase III	Randomized	132	Treatment	Ovarian cancer	Cisplatin	Not yet recruiting
NCT03371693	Phase III	Randomized	112	Treatment	Ovarian cancer	Lobaplatin	Active, not recruiting
NCT03717610	Phase III	N/A	10	Treatment	Ovarian cancer	Mitomycin C	Recruiting
NCT03842982	Phase III	Randomized	362	Treatment	Ovarian cancer	N/A	Recruiting
NCT04473339	Phase III	Randomized	280	Treatment	Ovarian cancer	N/A	Recruiting
NCT04111978	Phase III	Randomized	540	Treatment	Ovarian cancer	N/A	Recruiting
NCT01376752	Phase III	Randomized	415	Treatment	Recurrent ovarian cancer	Cisplatin	Active, not recruiting
NCT02158988	Phase III	Randomized	105	Treatment	Gastric cancer	Mitomycin C AND/OR Cisplatin	Completed
NCT02240524	Phase III	Randomized	582	Treatment	Gastric cancer	Paclitaxel	Unknown status
NCT02356276	Phase III	Randomized	584	Treatment	Gastric cancer	Paclitaxel	Unknown status
NCT02381847	Phase III	Randomized	60	Treatment	Gastric cancer	Cisplatin	Unknown status
NCT02960061	Phase III	Randomized	640	Treatment	Gastric cancer	Paclitaxel	Unknown status
NCT03179579	Phase III	Randomized	88	Treatment	Gastric cancer	Paclitaxel AND/OR Cisplatin	Not yet recruiting
NCT03917173	Phase III	Randomized	240	Treatment	Gastric cancer	Mitomycin C AND Cisplatin	Recruiting
NCT04447352	Phase III	Randomized	200	Treatment	Gastric cancer	Cisplatin	Recruiting
NCT04597294	Phase III	Randomized	600	Prevention	Gastric cancer	Irinotecan	Recruiting
NCT03772028	Phase III	Randomized	538	Treatment	Gastric cancer	Mitomycin C	Recruiting
NCT01882933	Phase III	Randomized	367	Treatment	Gastric cancer	Oxaliplatin	Active, not recruiting
NCT03023436	Phase III	N/A	220	Treatment	Gastric cancer	Cisplatin	Recruiting
NCT03348150	Phase III	Randomized	182	Treatment	Gastric cancer	Oxaliplatin AND Docetaxel	Recruiting
NCT02614534	Phase III	Randomized	200	Treatment	Colorectal cancer	Mitomycin C	Active, not recruiting
NCT02179489	Phase III	Randomized	300	Treatment	Colorectal cancer	Mitomycin C	Recruiting
NCT02965248	Phase III	Randomized	147	Treatment	Colorectal cancer	Oxaliplatin	Recruiting
NCT02974556	Phase III	Randomized	140	Prevention	Colorectal cancer	Oxaliplatin	Not yet recruiting
NCT03028155	Phase III	Randomized	60	Treatment	Colorectal cancer	Oxaliplatin	Unknown status
NCT03221608	Phase III	Randomized	300	Prevention	Colorectal cancer	Lobaplatin	Not yet recruiting
NCT03413254	Phase III	Randomized	389	Diagnostic	Colorectal cancer	N/A	Recruiting
NCT03914820	Phase III	Randomized	330	Treatment	Colorectal cancer	Mitomycin C	Recruiting
NCT04370925	Phase III	Randomized	688	Treatment	Colorectal cancer	Mitomycin C	Recruiting
NCT04861558	Phase III	Randomized	356	Treatment	Colorectal cancer	5 Fluorouracil AND/OR Irinotecan AND/OR Oxaliplatin	Recruiting
NCT03733184	Phase III	N/A	200	N/A	Colorectal cancer	Mitomycin C	Unknown status
NCT05250648	Phase IV	Randomized	216	Treatment	Colorectal cancer	Mytomicin C	Not yet recruiting

NR/NA: not reported or not applicable. Peritoneal metastases from each origin (from gastric, colorectal, appendiceal, hepatopancreatic, uterine or ovarian cancers or primary peritoneal tumors). Data gathered on 1 October 2022.

## Data Availability

The authors confirm that the data supporting the findings of this study are available within the article and its Supplementary Materials.

## Supplementary Materials

The following supporting information can be downloaded at: https://www.mdpi.com/article/10.3390/cancers15071926/s1, Table S1: Characteristics of HIPEC Trials already completed but not yet published results.
