# Supplementary material for: Current Research and Development in Hyperthermic Intraperitoneal Chemotherapy (HIPEC)—A Cross-Sectional Analysis of Clinical Trials Registered on ClinicalTrials.gov
Source: Cancers (Basel). 2023 Mar 23;15(7):1926. doi: 10.3390/cancers15071926 (PMC10093244; doi:10.3390/cancers15071926)
Supplement: Supplementary file 1 [file cancers-15-01926-s001.zip › cancers-2240758-supplementary.pdf]

cancers

MDPI

Supplementary Materials - Index

Supplementary Results

(…) 39 (20%) were completed but had not published results on the primary endpoint (shown in Supplementary Table S1). *Page7*

Supplementary Table

Table S1. Characteristics of HIPEC Trials already completed but not yet published results.

ClinicalTrials.gov Trial ID	Phase	Treatment allocation	Number of participants	Primary purpose of trial	Condition or disease	Chemotherapy drugs	Primary Outcome
NCT01226550	Phase 1/2	N/A	18	Treatment	Colorectal AND Gastric AND Primary peritoneal carcinom	Mitomycin C AND/OR Irinotecan	Safety
NCT01126346	N/A	N/A	10	Supportive care	Appendix AND Ovarian AND Colon AND Pseudomyxoma Peritonei AND Primary peritoneal carcinom	N/A	Safety
NCT02062749	Phase 1	N/A	1	Treatment	Colorectal cancer	Oxaliplatin	Pharmacodynamics / pharmacokinetics
NCT02219893	Phase 1/2	N/A	15	Treatment	Colorectal cancer	MOC31PE	Pharmacodynamics / pharmacokinetics
NCT02830139	Phase 2	Randomized	100	Treatment	Primary peritoneal carcinom AND Ovarian AND Tube carcinoma	Oxaliplatin AND/OR Capecitabine AND Cisplatin AND/OR 5-Fluorouracil	Efficacy
NCT03370588	N/A	Randomized	56	Supportive Care	Colorectal cancer	N/A	Efficacy
NCT03561948	N/A	Randomized	90	Prevention	Colorectal cancer	Mitomycin C	Feasibility
NCT01379482	Phase 2	N/A	18	Treatment	Gastric cancer	Cisplatin AND/OR Doxorubicin	Efficacy
NCT02158988	Phase 3	Randomized	105	Treatment	Gastric cancer	Mitomycin C AND/OR Cisplatin	Efficacy
NCT02528110	Phase 2	Randomized	100	Treatment	Gastric cancer	Paclitaxel AND/OR 5-Fluorouracil	Efficacy
NCT01854255	Phase 2	N/A	35	Treatment	Gastric cancer	Doxorubicin AND Cisplatin	Efficacy
NCT02903771	Phase 1	N/A	28	Treatment	Ovarian AND Tube carcinoma	Cantrixil	Feasibility AND Safety
NCT03931304	N/A	N/A	1200	N/A	Ovarian cancer	N/A	Efficacy
NCT04234243	N/A	N/A	144	N/A	Ovarian cancer	N/A	Efficacy
NCT00474669	Phase 1	Non-Randomized	30	Treatment	Ovarian cancer	Docetaxel	Pharmacodynamics / pharmacokinetics
NCT00001569	Phase 1	N/A	74	Treatment	Peritoneal Carcinomatosis from each origine	Cisplatin AND Paclitaxel AND 5-Fluorouracil	Efficacy
NCT00502177	N/A	N/A	48	N/A	Peritoneal Carcinomatosis from each origine	Cisplatin	Other
NCT01957852	N/A	N/A	86	N/A	Peritoneal Carcinomatosis from each origine	N/A	Other
NCT02082886	N/A	N/A	193	N/A	Peritoneal Carcinomatosis from each origine	N/A	Efficacy AND Safety
NCT02189434	N/A	N/A	20	N/A	Peritoneal Carcinomatosis from each origine	N/A	Other
NCT03430128	N/A	Randomized	70	Supportive Care	Peritoneal Carcinomatosis from each origine	N/A	Other
NCT03956836	N/A	N/A	46	N/A	Peritoneal Carcinomatosis from each origine	N/A	Other
NCT04083547	N/A	N/A	114	N/A	Peritoneal Carcinomatosis from each origine	N/A	Other
NCT04144465	N/A	Randomized	30	Treatment	Peritoneal Carcinomatosis from each origine	N/A	Other

NCT04664218	N/A	N/A	50	Other	Peritoneal Carcinomatosis from each origine	N/A	Other
NCT03895606	N/A	N/A	57	N/A	Peritoneal Carcinomatosis from each origine	N/A	Other
NCT04553900	N/A	N/A	28	Supportive Care	Peritoneal Carcinomatosis from each origine	N/A	Other
NCT04130347	N/A	N/A	499	N/A	Peritoneal Carcinomatosis from each origine	N/A	Other
NCT00436657	Phase 1	N/A	10	Treatment	Peritoneal Carcinomatosis from each origine	Cisplatin	Safety
NCT01812148	N/A	N/A	19	N/A	Primary peritoneal carcinom	Oxaliplatin	Efficacy
NCT02462564	N/A	N/A	31	N/A	Primary peritoneal carcinom	N/A	Other
NCT02374411	N/A	N/A	55	N/A	Primary peritoneal carcinom OR Appendiceal OR Pancreatic	N/A	Efficacy
NCT03034850	N/A	N/A	27	N/A	Colorectal OR Gastric OR Ovarian OR Primary peritoneal carcinom	N/A	Safety
NCT02040142	Phase 2	N/A	51	Treatment	Colorectal OR Gastric OR Appendiceal OR Primary peritoneal carcinom	Mitomycin C	Safety
NCT01709487	Phase 1/2	N/A	19	Treatment	Primary peritoneal carcinom OR Ovarian OR Tube	Cisplatin	Efficacy
NCT02199171	Phase 1	N/A	30	Treatment	Primary peritoneal carcinom OR Ovarian OR Tube	Carboplatin	Pharmacodynamics / pharmacokinetics
NCT02217956	Phase 1	N/A	30	Treatment	Primary peritoneal carcinom OR Ovarian OR Tube	Cisplatin	Pharmacodynamics / pharmacokinetics
NCT00625092	Phase 1	N/A	17	Treatment	Primary peritoneal carcinoma	5-Fluorouracil AND/OR Leucovorin AND/OR Oxaliplatin	Pharmacodynamics / pharmacokinetics
NCT02672865	Phase 1	N/A	4	Treatment	Gastric cancer	Mitomycin C AND Cisplatin	Feasibility AND Safety

NR/NA: not reported or not applicable; **Peritoneal metastases from each origin** (from gastric, colorectal, appendiceal, hepatopancreatic, uterine or ovarian cancers or primary peritoneal tumors). **Other**= Includes lab draws (inflammatory parameters, biomarkers etc.), tumoral biopsy tissus, microbiome, diagnostic test Data gathered on 01.10.2022.
